# Idiopathic Basal Ganglia Calcification Presented with Impulse Control Disorder

**DOI:** 10.1155/2015/287586

**Published:** 2015-07-13

**Authors:** Cem Sahin, Mustafa Levent, Gulhan Akbaba, Bilge Kara, Emine Nese Yeniceri, Betul Battaloglu Inanc

**Affiliations:** ^1^Department of Internal Medicine, School of Medicine, Mugla Sıtkı Kocman University, Orhaniye Mahallesi, İsmet Catak Caddesi, 48000 Mugla, Turkey; ^2^Department of Endocrinology, School of Medicine, Mugla Sıtkı Kocman University, Orhaniye Mahallesi, İsmet Catak Caddesi, 48000 Mugla, Turkey; ^3^Department of Psychiatry, School of Medicine, Mugla Sıtkı Kocman University, Orhaniye Mahallesi, İsmet Catak Caddesi, 48000 Mugla, Turkey; ^4^Department of Family Medicine, Faculty of Medicine, Mugla Sıtkı Kocman University, Orhaniye Mahallesi, İsmet Catak Caddesi, 48000 Mugla, Turkey

## Abstract

Primary familial brain calcification (PFBC), also referred to as Idiopathic Basal Ganglia Calcification (IBGC) or “Fahr's disease,” is a clinical condition characterized by symmetric and bilateral calcification of globus pallidus and also basal ganglions, cerebellar nuclei, and other deep cortical structures. It could be accompanied by parathyroid disorder and other metabolic disturbances. The clinical features are dysfunction of the calcified anatomic localization. IBGC most commonly presents with mental damage, convulsion, parkinson-like clinical picture, and neuropsychiatric behavior disorders; however, presentation with impulse control disorder is not a frequent presentation. In the current report, a 43-year-old male patient who has been admitted to psychiatry policlinic with the complaints of aggressive behavior episodes and who has been diagnosed with impulse control disorder and IBGC was evaluated in the light of the literature.

## 1. Introduction

The calcification of deep cortical structures and basal ganglions has been first defined histologically in 1855 by Bamberger. The disease is named by the name of German pathologist Karl Theodor Fahr who has first demonstrated the anatomical lesions. Fahr has first defined the disease in 1930 in an adult who had been under follow-up due to the symptoms of dementia and in which calcification had been detected in cerebral blood vessels in autopsy examination and has reported the disease as “idiopathic calcification in cerebral vessels” [1]. The radiological findings of the disease have been first defined by Modrego et al. in 2005 [2]. While the severity of calcification could be observed in direct roentgenograms, computerized tomography (CT) is a more sensitive modality than direct radiography in early diagnosis and demonstration of small calcifications.

Calcium deposits are histologically present in capillary vessels, media layer of small arteries, and vein and perivascular area. The disease is generally transmitted in an autosomal dominant pattern. Clinically Parkinsonism-like movement disorders, psychiatric symptoms, and radiologically nonatherosclerotic bilateral idiopathic calcification in basal ganglions are the necessary criteria for the diagnosis.

IBGC frequently begins between the fourth and the sixth decades. Generally calcium deposits appear in the first three decades of life and neurological impairment begins two decades later than calcium deposits. However, IBGC could rarely be observed in pediatric population also [3].

The patients with IBGC generally present with a clinical picture such as progressive mental damage, convulsion, Parkinson-like picture, and neuropsychiatric behavior disorders. However admission with impulse control disorder is not a frequently observed condition. In the present paper, a 43-year-old male patient who has been admitted to psychiatry policlinic with the complaints of aggressive behavior episodes and who has been diagnosed with impulse control disorder and idiopathic basal ganglia calcification was evaluated in the light of the literature.

## 2. Case Presentation

A 43-year-old male patient was evaluated in psychiatry policlinic due to the complaints of bursts of anger. From his medical history, it was learnt that his complaints have begun 5 years ago and have gradually increased within the last years. It was learnt that burst of anger has caused disturbance in social and professional life and important legal and financial problems and thus he wanted to have psychiatric support. The patient told that bursts of anger generally develop suddenly, continue for approximately half an hour, and include verbal, physical attacks and attacks against objects. He mentioned that he completely loses his control during burst of anger and it is not possible to oppose him and he is in a condition just like seizure and the severity of anger might be more or less independent of the stress causing the burst.

There was no feature in his medical and family history. As hypocalcemia was detected in laboratory examinations, he was consulted with the internal medicine department. On physical examination, general condition of the patient was normal; he was conscious and cooperated. Vital signs were normal (blood pressure 120/85 mm Hg, pulse 75 beat/min, respiratory rate 15/min, and body temperature: 36.8) and Chvostek test was (+) in his systemic and neurological examination. There was no additional pathology and the laboratory findings of the patient are summarized in Table 1.

**Figure 1:**
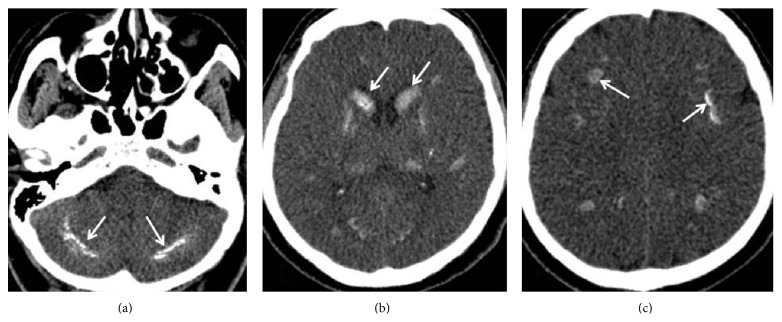
A large number of calcifications are observed in both cerebellar hemispheres (a), basal ganglions (b), and subcortical white matter (c) in axial CT sections (white arrows).

On posterior-anterior chest X-ray there was no pathological finding. ECG was in normal sinus rhythm. Computerized cranial tomography was performed for both differential diagnosis of hypocalcemia and differentiation of the burst of anger from an organic cause (Figure 1). There were a great number of calcifications in both cerebellar hemispheres (Figure 1(a)), basal ganglions (Figure 1(b)), and subcortical white matter (Figure 1(c)) in axial sections of cranial tomography. In the light of the present findings, the patient was diagnosed with idiopathic basal ganglia calcification.

Some tests were performed to exclude other diagnoses and VDRL test was negative; TORCH group Ig M was negative and Ig G was positive. Anti-HIV antibody was negative. No pathology was observed in ultrasonography of thyroid and parathyroid glands. Thyroid function tests were normal and thus hyperthyroidism and hypothyroidism were excluded. Vitamin D level was normal. As there were no previous infection, previous thyroid surgery, drug use, and autoimmune disease in detailed medical history of the patient, the hypocalcemia was thought to be caused by an idiopathic etiology. On psychiatric evaluation which was performed according to DSM IV criteria, the patient was diagnosed with impulse control disorder. With the present findings, the patient was accepted as IBGC and impulse control disorder. Intravenous and oral calcium replacement therapy was administered and carbamazepine 200 mg/dy was started by the psychiatry department. After normalization of calcium values the patient was discharged to be followed up in the policlinic.

## 3. Discussion

IBGC which is named as bilateral striopallidodentate calcinosis is a disease with unknown etiology and characterized with neurodegenerative disturbances developing after almost always bilateral accumulation of especially calcium and phosphor in basal ganglions, cerebellar dentate nucleus, and thalamus [4]. Although there is no certain information related to how the intracerebral calcifications develop, they are thought to be possibly related to mainly infectious diseases, metabolic and genetic disturbances [5]. In postmortem examinations of the patients with idiopathic basal ganglia calcification, calcifications are observed most commonly in cerebral sulcus, basal ganglion (especially globus pallidus), dentate nucleus, and subthalamus. The calcifications in adventitia and media layers of the vessels might surround all lumen and also it might be related to the intimal fibrosis and obstruction. The variability in extent of neuronal degeneration and gliosis is possibly related to the severity of ischemia.

The disease is slowly progressive. It is observed twofold more in males when compared with females. Most the patients demonstrate symptoms at the fourth and sixth decades. However, it is known that although rarely present, there are also cases reported in children in the literature [6].

Although IBGC seems to be related to many clinical conditions, there is still no consensus related to its etiology. Although, today, the most accepted opinion is development of the disease due to disturbances in calcium and phosphor metabolism, it has been demonstrated that the disease could also develop as a result of genetic damage without any change in calcium metabolism. The disease is generally inherited in autosomal dominant pattern [7]. But genetic heterogeneity also comes into question, because sporadic, familial, and autosomal recessive forms have also been reported. In a study which was conducted in a family in which this syndrome is observed, it has been demonstrated that a defect in short arm of 14th chromosome is important in development and progression of this disease [8].

The clinical symptoms have a wide range. Primarily neurological and psychiatric findings are observed in the patients. Among these the most common are Parkinsonism-like movement disorders and second common are cognitive disorders related to especially cerebellar involvement. While the conditions such as dystonia, tremor, chorea, ataxia, dementia, epilepsia, syncope, or stroke are frequently observed, behavioral disorders, personality changes, and several eye problems are observed at a lesser extent [9]. If the calcifications in the cranium are diffuse, other symptoms depending on the localization of the involved region could also develop. The most commonly involved basal ganglion is globus pallidus. The cause of many neuropsychiatric symptoms which could develop during the disease course is related to the anatomical localization of the globus pallidus and its relations.

In the literature it has been reported that neuropsychiatric symptoms are observed in patients with idiopathic basal ganglia calcification. There are reported cases which had been admitted with epilepsia, visual hallucinations, and sudden loss of consciousness. However, no case of IBGC accompanied with only impulse control disorder has been found in the literature review.

The most important parameter for diagnosis of IBGC is the presence of bilateral and symmetrical calcifications. An important part of these calcifications is accidentally recognized in computerized cranial tomography (CT) which is performed due to other causes. Among the radiological methods, as CT is more sensitive for calcifications, it is more valuable in diagnosis of IBGC when compared with magnetic resonance imaging (MR) [10]. In the presence of neuropsychiatric findings together with calcification in imaging methods as a supportive finding, idiopathic basal ganglia calcification should be considered in differential diagnosis if there is no other disease causing this clinical picture, because IBGC is an exclusion diagnosis.

Hypoparathyroidism and aged related physiological calcifications should be among the most commonly encountered differential diagnoses. Besides endocrinological causes such as pseudohypoparathyroidism, hypothyroidism, and D hypervitaminosis; infectious diseases such as toxoplasmosis, rubella, cytomegalovirus, HIV, and tuberculosis; vascular diseases such as Sturge-Weber disease; clinical situations related to toxic causes such as Wilson's disease, anoxia, carbon monoxide, and lead intoxication which are associated with calcifications in basal ganglions should also be considered [11].

There is no certain treatment method defined for FD in which the benefit has been demonstrated. Today the treatment is generally symptomatic. So the disease leads to progressive neurological impairment and death. However, calcium replacement therapy which is administered in the presence of hypocalcemia in addition to symptomatic treatment has been demonstrated to prevent the clinical progression of the disease. Because it has been demonstrated that long-term hypocalcemia increases the severity of calcifications in basal ganglions [12]. Furthermore, a calcium channel blocker, nimodipine which is specific to central nervous system, has been used together with regulation of calcium metabolism but successful results have not been obtained. It has been found that although disodium etidronate does not decrease calcifications, it provides symptomatic recovery [13]. Anticonvulsants have been used in cases accompanied with convulsions. Also cases have been reported in which ECT had beneficial effects on some psychotic symptoms.

## 4. Conclusion

Although IBGC has been known approximately for a century, it could be easily bypassed as it is rarely encountered in clinical practice.

IBGC should be considered in differential diagnosis of the patients admitted with the complaints of sudden impulse control disturbance who have calcium metabolism disturbance.

The cases with IBGC should be evaluated with laboratory tests assessing calcium metabolism and cranial CT.

## Figures and Tables

**Table 1:** Laboratory findings of the patient.

Parameters	Values
Sodium (mEq)	143
WBC (10^3^/mm^3^)	7300
Hb (mg/dL)	13.85
Platelets (/*μ*L)	240.000
Glucose (mg/dL)	65
BUN (mg/dL)	28
Creatinine (mg/dL)	0.82
ALT (U/L)	25
AST (U/L)	21
Folate (ng/mL)	9.63
Vitamin D (ng/mL)	43
Triglyceride (mg/dL)	494
Magnesium	1.64
Phosphor (U/L)	3.8
Albumin (gr/dL)	4.44
Calcium (mg/dL)	6.53
Ionized calcium (mg/dL)	2.86
PTH (pg/m)	21
fT3 (pmol/L)	4.96
fT4 (pmol/L)	16.62
TSH (*μ*IU/m)	0.899
vitB12 (pg/mL)	229.2
Cholesterol (mg/dL)	222
VLDL (mg/dL)	99
